# Risk factors for disease severity among children with Covid-19: a clinical prediction model

**DOI:** 10.1186/s12879-023-08357-y

**Published:** 2023-06-12

**Authors:** David Chun-Ern Ng, Chuin-Hen Liew, Kah Kee Tan, Ling Chin, Grace Sieng Sing Ting, Nur Fadzreena Fadzilah, Hui Yi Lim, Nur Emylia Zailanalhuddin, Shir Fong Tan, Muhamad Akmal Affan, Fatin Farihah Wan Ahmad Nasir, Thayasheri Subramaniam, Marlindawati Mohd Ali, Mohammad Faid Abd Rashid, Song-Quan Ong, Chin Chin Ch’ng

**Affiliations:** 1 Hospital Tuanku Ja’afar, Negeri Sembilan, Ministry of Health, Jalan Rasah, 70300 Seremban, Malaysia; 2 Hospital Tuanku Ampuan Najihah, Negeri Sembilan, Ministry of Health, Jalan Melang, 72000 Kuala Pilah, Malaysia; 3 Perdana University Seremban Clinical Academic Center, Negeri Sembilan, Jalan Rasah, 70300 Seremban, Malaysia; 4 Negeri Sembilan State Health Department, Negeri Sembilan, Ministry of Health, Jalan Rasah, 70300 Seremban, Malaysia; 5 Institute for Tropical Biology and Conservation, University Malaysia Sabah, Jalan UMS, 88400 Kota Kinabalu, Sabah, Malaysia; 6 Clinical Research Centre Hospital Pulau Pinang, Ministry of Health, Jalan Residensi, 10450, Pulau Pinang, Malaysia

**Keywords:** COVID-19, Pediatric, Nomogram, Predictor severity

## Abstract

**Background:**

Children account for a significant proportion of COVID-19 hospitalizations, but data on the predictors of disease severity in children are limited. We aimed to identify risk factors associated with moderate/severe COVID-19 and develop a nomogram for predicting children with moderate/severe COVID-19.

**Methods:**

We identified children ≤ 12 years old hospitalized for COVID-19 across five hospitals in Negeri Sembilan, Malaysia, from 1 January 2021 to 31 December 2021 from the state’s pediatric COVID-19 case registration system. The primary outcome was the development of moderate/severe COVID-19 during hospitalization. Multivariate logistic regression was performed to identify independent risk factors for moderate/severe COVID-19. A nomogram was constructed to predict moderate/severe disease. The model performance was evaluated using the area under the curve (AUC), sensitivity, specificity, and accuracy.

**Results:**

A total of 1,717 patients were included. After excluding the asymptomatic cases, 1,234 patients (1,023 mild cases and 211 moderate/severe cases) were used to develop the prediction model. Nine independent risk factors were identified, including the presence of at least one comorbidity, shortness of breath, vomiting, diarrhea, rash, seizures, temperature on arrival, chest recessions, and abnormal breath sounds. The nomogram’s sensitivity, specificity, accuracy, and AUC for predicting moderate/severe COVID-19 were 58·1%, 80·5%, 76·8%, and 0·86 (95% CI, 0·79 – 0·92) respectively.

**Conclusion:**

Our nomogram, which incorporated readily available clinical parameters, would be useful to facilitate individualized clinical decisions.

**Supplementary Information:**

The online version contains supplementary material available at 10.1186/s12879-023-08357-y.

## Introduction

Coronavirus disease 2019 (COVID-19), caused by severe acute respiratory syndrome coronavirus 2 (SARS-CoV-2), presents in a spectrum with varying severity across different age groups of patients. Although children generally experience a mild clinical course of illness, some develop severe disease requiring hospitalization or admission to intensive care units [1]. COVID-19 has many clinical manifestations in children, ranging from asymptomatic infection to upper respiratory tract symptoms, febrile seizures, gastrointestinal symptoms, and severe pneumonia [2].

Most studies on the risk factors for severity of pediatric COVID-19 have focused on children from the United States and Europe [3–5]. The Southeast Asian region has experienced high rates of COVID-19 infection and bears a significant burden of the disease worldwide. There is a need for representative clinical data from children in Southeast Asia, where differences in age distribution, comorbidities, access to healthcare services, and other factors could influence the severity and clinical outcomes. Furthermore, clinical prediction model-based studies in children to define COVID-19 severity are lacking.

In this study, we first describe the epidemiological and clinical characteristics of COVID-19 in children ≤ 12 years old who were hospitalized for COVID-19 in the state of Negeri Sembilan, Malaysia, over a 12-month period in 2021. We evaluated risk factors for moderate/severe disease among children with SARS-CoV-2 infection and constructed a nomogram using common clinical features to predict children who would require medical intervention during hospitalization. By employing the nomogram to stratify children according to the likelihood of moderate/severe disease, clinicians could prioritize their patients and optimize medical resources.

## Methods

### Setting and design

The study period encompasses an initial phase of the pandemic, where all children with COVID-19 required hospital isolation as part of the country’s containment measures before home quarantine was introduced for suitable patients [6]. Patients with COVID-19 were admitted across five designated hospitals within the state depending on their severity, and their data were recorded in an online case registration system. This digital registry captured demographics, clinical features, and laboratory results of the patients and was utilized for hospital bed management in the five hospitals, comprising a single tertiary hospital and four district hospitals. Since pediatric isolation beds in the tertiary hospital were limited, the registry was used to assist with decisions to step up care to the tertiary hospital or to step down care to district hospitals based on the severity of the symptoms. The five hospitals in the state served approximately 1,100,000 people, including 215,000 children aged ≤ 12 years. This study was performed before COVID-19 vaccinations were available in the country for children below 12 years old.

We identified children ≤ 12 years old hospitalized with laboratory-proven SARS-CoV-2 infection between 1 January 2021 to 31 December 2021 from the state’s pediatric infectious disease case registration system. Neonatal patients tested positive within the first 48 h of birth and children hospitalized with multisystem inflammatory syndrome (MIS-C) were excluded from this study.

### Study definitions

A laboratory-confirmed case was defined as a positive reverse transcription-polymerase chain reaction (RT-PCR) or rapid antigen detection result from respiratory samples (combined oro/ nasopharyngeal swab or endotracheal aspirate from ventilated patients). Fever was defined as a body temperature ≥ 37·5 °C. Chest recessions may be intercostal, subcostal, sternal, or suprasternal. Abnormal breath sounds were defined as the presence of stridor or crackles, wheeze, or rhonchi during lung auscultation. Patients were categorized as having obesity based on reporting by clinicians or if they had a body mass index ≥ 95^th^ centile for their age and sex [7].

We adapted the World Health Organization (WHO) ordinal scale to categorize the degree of COVID-19 severity, which reflected the disease severity and resource utilization over the course of the clinical illness [8]. The WHO ordinal scale classifies COVID-19 severity into five categories: uninfected, ambulatory mild disease, hospitalized moderate disease, hospitalized severe disease, or dead. In this ordinal scale, symptomatic patients hospitalized only for isolation purposes are classified as having mild disease. For simplification, we categorized the patients into the following four categories:

- Asymptomatic: Asymptomatic patients who were hospitalized for isolation purposes.
- Mild disease: Symptomatic patients who were hospitalized but did not require any medical intervention apart from clinical surveillance.
- Moderate disease: Symptomatic patients who were hospitalized and required medical interventions such as intravenous fluids, steroids, oxygen by mask or nasal prong, empirical antibiotics, or blood investigation monitoring. There were no manifestations related to severe disease.
- Severe disease: Features of moderate disease, plus any manifestations that suggest disease progression, such as respiratory distress needing oxygen support via high-flow nasal cannula, non-invasive ventilation, or mechanical ventilation, with or without inotropes. Patients requiring pediatric ICU (PICU) care were also considered to have severe disease. Patients were admitted to the PICU if they required inotropes, non-invasive ventilation, mechanical ventilatory support, or continuous vital sign monitoring based on the clinician’s discretion.

### Data management and statistical analysis

The registry was accessed to extract patient demographics, comorbidities, presenting symptoms, physical signs, laboratory parameters, treatment received, length of stay, and outcomes, which were then transferred to a Microsoft Excel spreadsheet for cleaning and coding. The cleaned data was exported to SPSS version 26.0 (IBM Corp., Armonk, NY, USA) for statistical analyses. Statistical significance was set at *p* < 0·05.

**Fig. 1:**
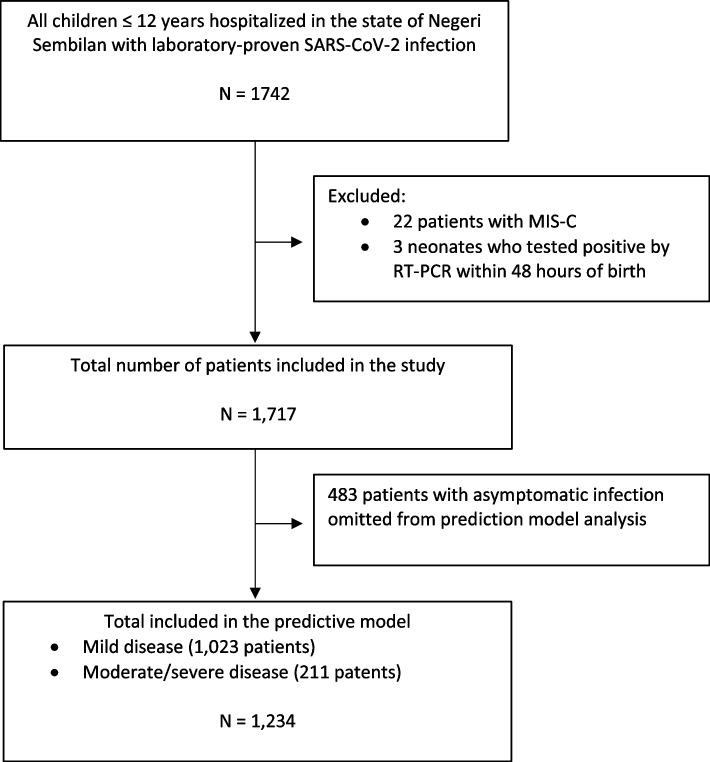
Flow diagram depicting the selection of study patients

Categorical variables were expressed as frequencies and percentages (%), whereas continuous variables were expressed as medians and interquartile ranges (IQR). Chi-squared or Fisher’s exact tests were used to compare categorical variables, and Mann–Whitney U tests were used to compare continuous variables, where appropriate. Disease severity was analyzed as a binary outcome whereby moderate and severe disease were combined into a single entity to reflect the need for medical intervention during hospitalization. Patients with mild disease served as a control group for comparative analyses. Asymptomatic patients hospitalized solely for isolation were excluded from the severity analyses (Fig. 1). Variables with missing data were excluded from the analysis; these included laboratory and radiological imaging results. All other variables had no missing values, so no imputation was required.

Statistical filter methods (Chi-squared test, Mann–Whitney U test) with SPSS were used to select significant variables for logistic regression model building. Variables that remained significantly associated (*p* < 0·05) with moderate/severe disease in bivariate statistical analysis (Mann–Whitney U Test, Chi-squared test, Fisher's exact test, univariate logistic regression) were included in the multivariate logistic regression to identify independent factors associated with disease severity. Odds ratios (ORs) and 95% confidence intervals (CIs) were calculated. The final prediction model included factors that remained significant with a *p*-value < 0·05. Multicollinearity between independent variables was assessed using a correlation matrix. Independent variables with a correlation of more than 0·7 were considered to have high collinearity and were excluded from the prediction model.

The data were randomly divided with a split-sample approach, allocating 80% of the data for training and 20% for internal validation. The model development and internal validation process are further explained in Supplementary Fig. 1. A nomogram was developed to visualize the logistic regression model and to enhance its clinical applicability. The nomogram was built using the rms package in R (version 4·1·1). Each variable was assigned a score according to the regression coefficients in the multivariate logistic regression. The total score varied from 0 to 160 and was computed by summing the individual scores for each parameter. To assess the performance of the nomogram in predicting SARS-CoV-2 severity, the trained model was tested on the validation cohort, which remained unexposed to the data. The model performance was evaluated using the area under the curve (AUC), sensitivity, specificity, and accuracy. We assessed goodness of fit through the Hosmer–Lemeshow (HL) test, where an HL test *p* ≥ 0·05 indicated the nomogram showed a good fit.

### Ethical considerations

The study was reviewed and approved by the Medical Research and Ethics Committee, Ministry of Health Malaysia [NMRR-22–00977-CQB(2)] and received informed consent exemption. No personal or identifiable data were collected during the conduct of the study.

## Results

**Fig. 2:**
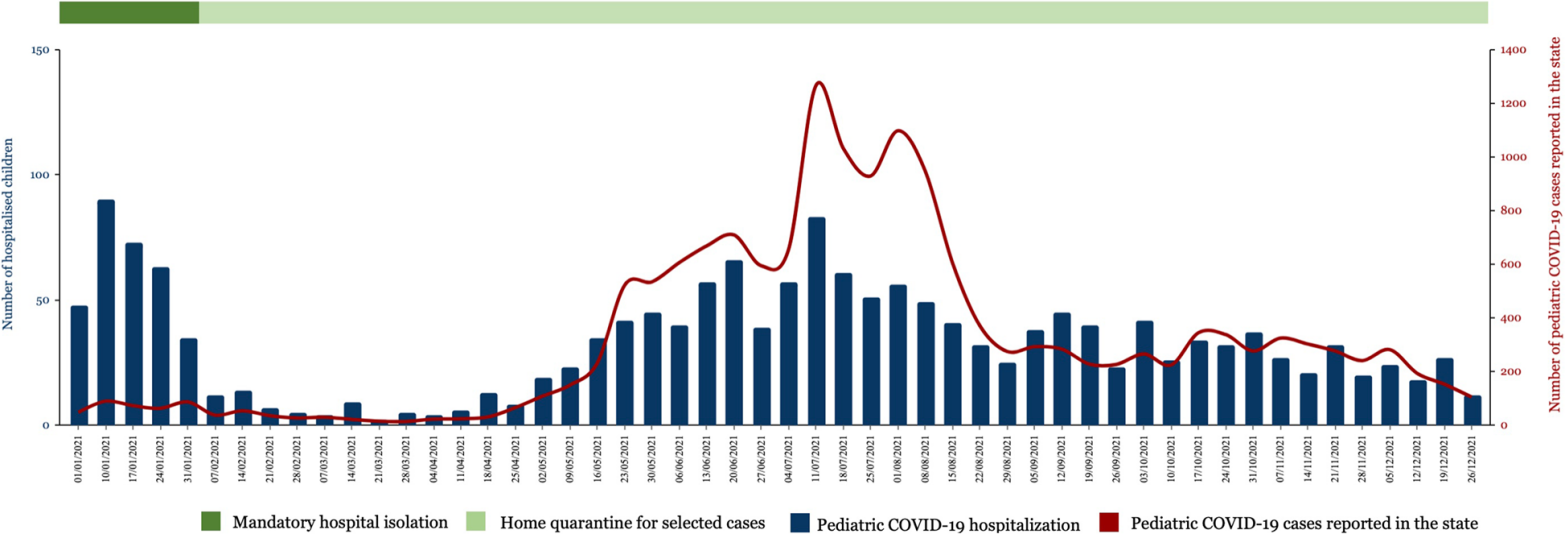
Trends of hospitalization of pediatric COVID-19 in relation to the number of pediatric COVID-19 cases reported in the state, January – December 2021. Data for pediatric COVID-19 cases reported in the state was obtained from the state health department

We identified 1,717 hospitalized pediatric patients with laboratory-confirmed SARS-CoV-2 infection during the 12-month study period. The high number of hospitalizations in the first four weeks of the year occurred in the setting of mandatory hospital isolation for all confirmed COVID-19 cases (Fig. 2). Subsequently, hospitalization rates decreased as home quarantine was implemented for suitable patients. The peak of hospitalizations occurred from mid-July to early August 2021, in tandem with the rise of pediatric COVID-19 cases in the state. The baseline characteristics of the study population are shown in Table 1. The median age of the patients was 3·2 years (IQR 1·1 – 7·7), and 51·5% were male. At least one comorbidity was identified in 142 patients (8·3%), with respiratory conditions being the most commonly reported comorbidity. Data on prematurity (defined as birth before completion of 37 weeks’ gestation) were collected only for children aged under two years, and 34 (2·0%) were premature. Clinical severity was classified as asymptomatic in 483 (28·1%) patients, mild in 1023 (59·6%) patients, moderate in 180 (10·5%) patients, and severe in 31 (1·8%) patients. Thirty-one (1·8%) patients required PICU care, indicating a PICU admission rate of 18 per 1,000 hospitalized children with COVID-19, or 2 per 1,000 children diagnosed with pediatric COVID-19 in the state. No mortalities were recorded during the study period.

**Table 1:** Baseline characteristics of the study population

Baseline Characteristics of Patients	Total (*n* = 1,717)
**Age in years, median (IQR)**	3·2 (1·1 – 7·7)
**• < 1 year**	408 (23·8%)
**• 1–5 years**	745 (43·4%)
**• > 5 years**	564 (32·8%)
**Male sex**	884 (51·5%)
**Comorbidities**^a^	
**• None**	1575 (91·7%)
**• Respiratory**	49 (2·9%)
**• Prematurity (if age < 2 years old)**^**b**^	34 (2·0%)
**• Cardiovascular**	30 (1·7%)
**• Genetic disorders**	18 (1·0%)
**• Neuromuscular**	15 (0·9%)
**• Others**	20 (1·2%)
**Symptoms on Presentation**^c^	
**• Asymptomatic**	483 (28·1%)
**• Fever**	972 (56·6%)
**• Cough**	508 (29·6%)
**• Rhinorrhea**	442 (25·7%)
**• Vomiting**	117 (6·8%)
**• Diarrhea**	98 (5·7%)
**• Shortness of breath**	53 (3·1%)
**• Anosmia/ageusia**	47 (2·7%)
**• Sore throat**	41 (2·4%)
**• Rashes**	33 (1·9%)
**• Seizures**	33 (1·9%)
**Treatment Received**^d^	
**• Intravenous fluids**	62 (3·6%)
**• Antibiotics**	66 (3·8%)
**• Steroids**	23 (1·3%)
**• Respiratory support**	62 (3·6%)
**◦ Low-flow nasal oxygen**	53 (3·1%)
**◦ High-flow nasal cannula/non-invasive ventilation**	8 (0·5%)
**◦ Mechanical ventilation**	1 (0·1%)
**Clinical Severity**	
**• Asymptomatic**	483 (28·1%)
**• Mild disease**	1023 (59·6%)
**• Moderate disease**	180 (10·5%)
**• Severe disease**	31 (1·8%)
**Clinical Outcomes**	
**• Pediatric intensive care unit (PICU) admission**	31 (1·8%)
**• Length of stay in days, median (IQR)**	3 (2–6)
**• Discharged alive**	1717 (100%)

*IQR* Interquartile range

^a^A patient may have more than one comorbidity

^b^Gestational age < 37 weeks at birth among children aged < 2 years

^c^Some patients presented with more than one symptom

^d^Some patients received more than one treatment

The most common presenting symptoms were fever (56·6%), followed by cough (29·6%), and rhinorrhea (25·7%). We grouped the patients into eight common phenotypes encountered in clinical practice (Table 2). Among asymptomatic patients, there was a higher proportion of older children aged 6 to 12 compared with children aged 0 to 5 years old (32·8% vs 25·8%, *p* = 0·003). Symptomatic children presented with two major clinical phenotypes: upper respiratory tract infection or viral fever with non-specific symptoms. The clinical phenotypes of COVID-19 varied between children aged 0 to 5 years and 6 to 12 years old. Children in the younger age group were more likely to present with fever with non-specific symptoms (OR 1·47; 95% CI 1·14 – 1·90), lower respiratory tract infection (OR 3·09; 95% CI 1·52 – 6·30), febrile seizures (OR 3·61; 95% CI 1·26 – 10·33) and acute gastroenteritis (OR 2·23, 95% CI 1·24 – 4·02).

**Table 2:** Clinical phenotypes of COVID-19 based on age group

	**Total** **(*n* = 1,717)**	**0 to 5 years old** **(*n* = 1,153)**	**6 to 12 years old** **(*n* = 564)**	***p*-value**	**Odds ratio** **(95% CI)**^a^
**Asymptomatic**	483 (28·1%)	298 (25·8%)	185 (32·8%)	0·003	0·71 (0·57 – 0·89)
**Fever with non-specific symptoms**^b^	381 (22·2%)	280 (24·3%)	101 (17·9%)	0·003	1·47 (1·14 – 1·90)
**Upper respiratory tract infection**	670 (39·0%)	424 (36·7%)	246 (43·6%)	0·006	0·75 (0·61 – 0·92)
**Croup**	2 (0·1%)	2 (0·2%)	0	1·0	-
**Exacerbation of asthma**	8 (0·5%)	3 (0·3%)	5 (0·9%)	0·07	0·29 (0·07 – 1·23)
**Lower respiratory tract infection**	64 (3·7%)	55 (4·8%)	9 (1·6%)	0·001	3·09 (1·52 – 6·30)
**Febrile seizures**	33 (2·0%)	29 (2·5%)	4 (0·7%)	0·01	3·61 (1·26 – 10·33)
**Acute gastroenteritis**	76 (4·4%)	62 (5·4%)	14 (2·5%)	0·006	2·23 (1·24 – 4·02)

*CI* confidence interval

^a^Using 6 – 12 years old patients as the reference group

^b^Non-specific symptoms include headache, myalgia, fatigue, rashes, red eyes, and vomiting without diarrhea

## Comparison of clinical characteristics between patients with mild illness and moderate/severe illness

Patient characteristics between mild and moderate/severe groups were compared in Table 3. The median age of patients with moderate/severe disease (1·9 years, IQR 0·7 – 6·2) was significantly younger than those with mild disease (3·1 years, IQR 1·0 – 7·6, *p* < 0·001). A higher proportion of males was observed in the moderate/severe group (57·3% vs 49·9%, *p* = 0·05). The presence of comorbidities was significantly higher in the moderate/severe group (18·5% vs 7·5%, *p* < 0·001). Obesity appeared to be higher in the moderate/severe group; however, it was not statistically significant (6·2% vs 3·4%, *p* = 0·06).

**Table 3:** Comparison of clinical features among patients with mild and moderate/severe disease

	**Mild Disease** **(*n* = 1,023)**	**Moderate/severe Disease** **(*n* = 211)**	***p*-value**
**Demography**			
Age in years, median (IQR)	3·1 (1·0 – 7·6)	1·9 (0·7 – 6·2)	< 0·001^c^
Male sex	511 (49·9%)	121 (57·3%)	0·05^d^
Malay ethnicity	844 (82·5%)	183 (86·7%)	0·09^d^
**Previous COVID-19 infection**^a^	6 (0·6%)	0 (0%)	0·60^d^
**Obesity**	35 (3·4%)	13 (6·2%)	0·06^d^
**Any comorbidities**	77 (7·5%)	39 (18·5%)	< 0·001^d^
**Type of Comorbidities**			
Respiratory	34 (3·3%)	11 (5·2%)	0·18^d^
Prematurity (if age < 2 years)^b^	21 (2·1%)	7 (3·3%)	0·26^d^
Cardiovascular	15 (1·5%)	9 (4·3%)	0·01^d^
Genetic	10 (1·0%)	6 (2·8%)	0·03^d^
Neuromuscular	7 (0·7%)	8 (3·8%)	< 0·001^d^
Gastrointestinal	2 (0·2%)	4 (1·9%)	0·001^e^
Others	10 (1·0%)	4 (1·9%)	0·25^d^
**Symptoms**			
Fever	793 (77·5%)	178 (84·4%)	0·03^d^
Cough	419 (41·0%)	88 (41·7%)	0·84^d^
Rhinorrhea	377 (36·9%)	65 (30·8%)	0·10^d^
Sore throat	36 (3·5%)	5 (2·4%)	0·40^d^
Shortness of breath	10 (1·0%)	43 (20·4%)	< 0·001^d^
Vomiting	65 (6·4%)	52 (24·6%)	< 0·001^d^
Diarrhea	63 (6·2%)	35 (16·6%)	< 0·001^d^
Seizures	11 (1·1%)	22 (10·4%)	< 0·001^d^
Rash	22 (2·2%)	11 (5·2%)	0·01^d^
Anosmia/ageusia	44 (4·3%)	3 (1·4%)	0·05^d^
**Physical Findings**			
Temperature on arrival in ºC, median (IQR)	36·6 (36·4 – 36·8)	37·0 (36·8 – 38·0)	< 0·001^c^
Chest recessions	2 (0·2%)	42 (19·9%)	< 0·001^d^
Abnormal breath sounds	3 (0·3%)	21 (10·0%)	< 0·001^d^

*IQR* Interquartile range

^a^A documented history of COVID-19 more than 3 months prior to the current hospitalization

^b^Gestational age < 37 weeks at birth among children aged < 2 years

^c^Mann-Whitney U Test,

^d^Chi-squared test

^e^Fisher's exact test

Patients with moderate/severe disease had a significantly higher proportion of fever (84·4% vs 77·5%, *p* = 0·03), shortness of breath (20·4% vs 1·0%, *p* < 0·001), vomiting (24·6% vs 6·4%, *p* < 0·001), diarrhea (16·6% vs 6·2%, *p* < 0·001), rashes (5·2% vs 2·2%, *p* = 0·01), and seizures (10·4% vs 1·1%, *p* < 0·001). Conversely, a higher proportion of patients with anosmia/ageusia were observed in the mild group (4·3% vs 1·4%, *p* = 0·05). A higher median temperature on arrival at the hospital was observed in the moderate/severe group compared with the mild group (37·0 °C vs 36·6 °C, *p* < 0·001). Additionally, the proportion of patients with documented chest recessions (19·9%), and abnormal breath sounds (10·0%) were significantly higher in patients with moderate/severe disease compared to those with mild disease (0·2%, *p* < 0·001; 0·3%, *p* < 0·001, respectively).

### Prediction model for moderate/severe COVID-19 and nomogram construction

Multivariate logistic regression analysis in Table 4 identified nine independent predictors of moderate/severe COVID-19: presence of at least 1 comorbidity (aOR 2·03, 95% CI 1·12 – 3·66, *p* = 0·02), shortness of breath (aOR 8·70, 95% CI 3·43 – 22·06, *p* < 0·001), vomiting (aOR 3·32, 95% CI 1·99 – 5·55, *p* < 0·001), diarrhea (aOR 2·87, 95% CI 1·61 – 5·09, *p* < 0·001), rashes (aOR 2·58, 95% CI 1·03 – 6·46, *p* = 0·04), seizures (aOR 4·47, 95% CI 1·79 – 11·19, *p* = 0·001), a higher temperature on arrival (aOR 2·88 per 1 °C increase, 95% CI 2·30 – 3·63, *p* < 0·001), chest recessions (aOR 28·84, 95% CI 5·95 – 139·69, *p* < 0·001), and abnormal breath sounds (aOR 5·89, 95% CI 1·09 – 31·76, *p* = 0·04). No significant collinearity was observed between the independent variables (Supplementary Fig. 2).

**Table 4:** Predictors of moderate/severe disease among hospitalized children with COVID-19

	**Univariate Logistic Regression**		**Multivariate Logistic Regression**	
	OR (95% CI)	*p*-value	aOR (95% CI)	*p*-value
**Age (years)**^a^	0·94 (0·90 – 0·98)	0·003	0·96 (0·90 – 1·01)	0·10
**Presence of any comorbidity**	2·79 (1·83 – 4·23)	< 0·001	2·03 (1·12 – 3·66)	0·02
**Fever**	1·56 (1·05 – 2·33)	0·03	1·32 (0·75 – 2·35)	0·34
**Shortness of breath**	25·93 (12·78 – 52·59)	< 0·001	8·70 (3·43 – 22·06)	< 0·001
**Vomiting**	4·82 (3·23 – 7·20)	< 0·001	3·32 (1·99 – 5·55)	< 0·001
**Diarrhea**	3·03 (1·95 – 4·72)	< 0·001	2·87 (1·61 – 5·09)	< 0·001
**Rash**	2·50 (1·20 – 5·24)	0·02	2·58 (1·03 – 6·46)	0·04
**Seizures**	10·71 (5·11 – 22·45)	< 0·001	4·47 (1·79 – 11·19)	0·001
**Temperature on arrival (°C)**^a^	3·32 (2·74 – 4·04)	< 0·001	2·88 (2·30 – 3·63)	< 0·001
**Chest recessions**	126·87 (30·43 – 529·00)	< 0·001	28·84 (5·95 – 139·69)	< 0·001
**Abnormal breath sounds**	37·58 (11·10 – 127·24)	< 0·001	5·89 (1·09 – 31·76)	0·04

*OR* odds ratio, *aOR* adjusted odds ratio, *CI* confidence interval

^a^per 1-unit increase

**Fig. 3:**
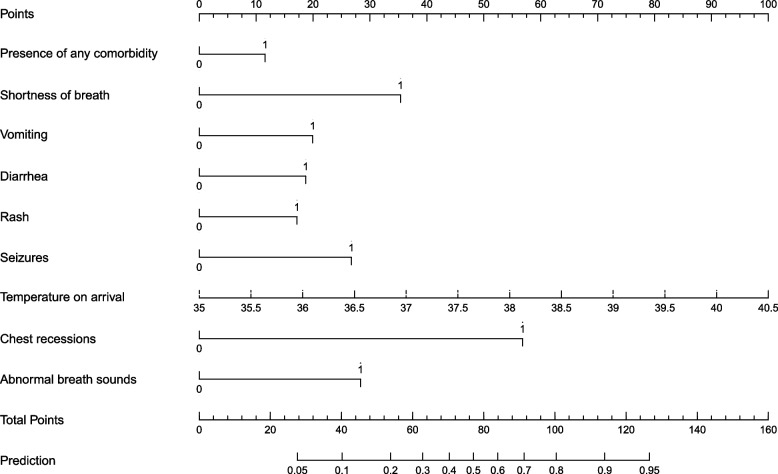
Nomogram predicting the probability of moderate to severe COVID-19. The nomogram was based on nine predictors found significant in the multivariate logistic regression (see results section). The weight of each variable was determined based on the regression coefficient. To use the nomogram, a score is assigned to each variable by drawing a line upward to the "points" axis. The total score is determined by adding the values of the nine variables. The probability of severe SARS-CoV-2 infection can be estimated by drawing a straight line from the total points axis (0 to 160) to the prediction axis

**Fig. 4:**
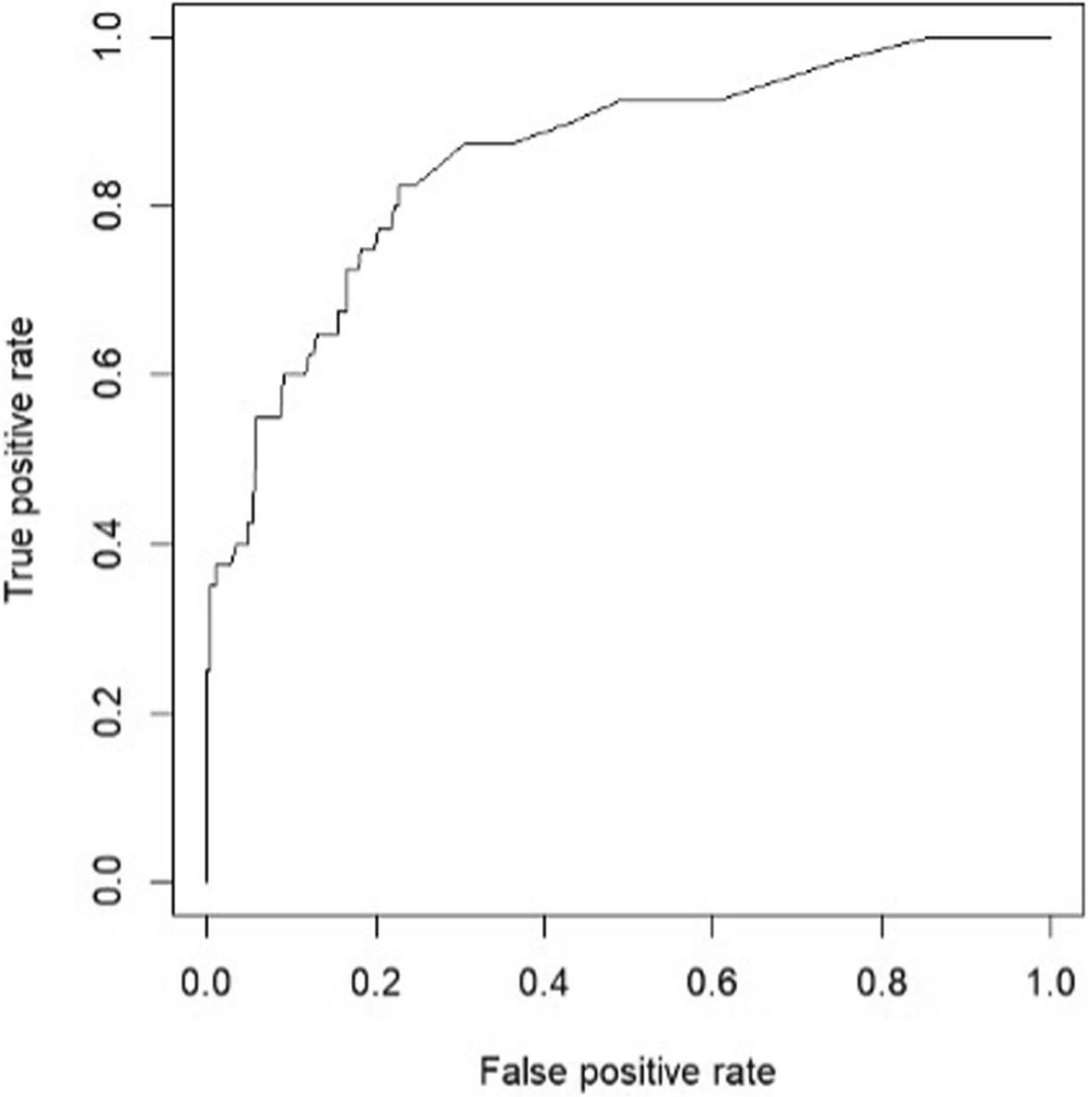
Receiver operating characteristic (ROC) curve of the nomogram predicting moderate/severe COVID-19. The validation of discrimination power of the nomogram was evaluated using ROC curve analysis. The y-axis represents the true positive rate, the x-axis represents the false positive rate, and the area under the curve (AUC) measures the discriminative power

A logistic regression model for predicting the probability of moderate/severe COVID-19 was developed and illustrated using a nomogram (Fig. 3). The nomogram was constructed using the nine independent variables associated with moderate/severe COVID-19. The temperature on arrival and the presence of chest recessions had the highest weight in predicting moderate/severe COVID-19. The model’s performance achieved an area under the curve (AUC) of 0·86 (95% CI 0·79 – 0·92, Fig. 4), indicating good discrimination of mild COVID-19 from moderate/severe disease. The nomogram’s sensitivity was 58·1% [standard deviation (SD) ± 27·3%], specificity was 80·5% (SD ± 25·8%), and accuracy was 76·8% (SD ± 18·4%). Our nomogram’s HL test *p*-value was 0·05, indicating an acceptable fit for the prediction model.

## Discussion

We described the epidemiology, clinical manifestations, and risk factors for the severity of pediatric COVID-19 based on a multicenter statewide study and developed a nomogram to predict the need for hospitalization. The study coincided with the period when the Delta variant predominated in the country [9].

Our study recorded the hospitalization trends with the statewide epidemic curve of pediatric COVID-19 in 2021. Home quarantine measures have yet to be introduced at the start of the year, explaining the high number of children hospitalized, even for asymptomatic/mild infections. COVID-19 vaccination in the adult population began in the late first quarter of 2021, covering only 20% of adults at the peak of the epidemic curve in July (Supplementary Fig. 3). Consequently, some children were hospitalized because they accompanied their parents, who were hospitalized for severe illnesses. Most children were diagnosed with COVID-19 after exposure to an infected adult within the household [10].

The criteria for hospitalization were dynamic as the pandemic evolved. The circumstances in 2021 resulted in two diverse groups of children being hospitalized: one group for non-medical reasons (mandatory hospital isolation or accompanying parents who required hospitalization) and another group of patients hospitalized due to disease severity. This presented the opportunity to study parameters that predict disease severity by comparing children who needed hospital care against a baseline group of hospitalized patients without medical interventions. The epidemiological data over the 12 months was necessary to understand the context and the definitions of disease severity used in this study.

The definition of severity for COVID-19 varies considerably in literature, with hypoxia as a common denominator for severe disease [11, 12]. Some studies included signs of dehydration as part of the diagnostic criteria [13, 14], whereas others defined severe disease as requiring ICU care [15]. Our study’s disease severity was adopted from the WHO clinical progression scale, [8] which recognized that patients might be hospitalized solely for isolation purposes and accommodated this factor in the definition of severity. The PICU admission rates reported in previous studies varied widely from 3·5% to 28% [5, 16, 17]. However, our study’s proportion of patients admitted to intensive care was likely spuriously low, considering the substantial number of children hospitalized for asymptomatic/mild disease and the exclusion of patients with MIS-C. Patients with MIS-C were not part of the inclusion criteria despite some requiring admission to the PICU as MIS-C is a distinct clinical entity from acute SARS-CoV-2 infection. The PICU admission rate of 2 per 1,000 diagnosed pediatric COVID-19 cases in the state was a more appropriate indicator of the disease burden in our setting. Notably, there were no mortalities recorded throughout the year. Our findings were consistent with the low mortality rates in children from previous reports [18, 19] and the low number of pediatric deaths recorded over two years of the pandemic in Malaysia [20].

COVID-19 has mainly been described as a respiratory illness early in the pandemic [11, 14]. However, the awareness of its clinical manifestations has evolved as the pandemic progressed [21, 22]. We categorized pediatric COVID-19 into clinical phenotypes to account for the common syndromic diagnosis in clinical practice. The substantial proportion of asymptomatic patients in our study was not unusual, given that much higher rates of asymptomatic infection have been detected previously when strict contact tracing and containment measures encompassed the entire spectrum of pediatric COVID-19 in the community [23]. In addition, we observed that febrile seizures or acute gastroenteritis might be the initial and primary manifestations of COVID-19 in children. Viral croup was an uncommon manifestation during this study period, but cases have increased sharply with the emergence of the Omicron variant [24].

Our results revealed nine clinical parameters that were independent risk factors for moderate/severe disease, including vomiting, diarrhea, rashes, shortness of breath, seizures, chest recessions, abnormal breath sounds, the temperature on arrival, and the presence of at least one comorbidity. Patients with asymptomatic infection were excluded from the multivariate analysis to avoid ascertainment bias. Extremes of age, such as young infants and adolescents, were found to be at higher risk for severe illness in past studies [25, 26]. However, age was not a significant predictor of severity in our study. A possible explanation for this finding is that more than three-quarters of our cohort were above the infancy age group, and adolescents were excluded from this study.

Dyspnea was a significant risk factor for severe COVID-19 in children [25, 27]. In agreement with previous studies, our findings revealed an 8·7-fold and 28·8-fold increase in the odds of moderate/severe illness for patients with shortness of breath and chest recessions, respectively. We demonstrated that the presence of any comorbidities was predictive of moderate/severe illness. This concurs with existing literature showing that underlying comorbidities have an important effect on the outcomes of pediatric COVID-19, particularly pulmonary disease, neurologic disorders, cardiovascular disease, and obesity [3, 4]. However, we classified comorbidities as either present or absent rather than individually, which was more useful in the clinical setting. Seizures were a predictor of severity in our study. In a previous study of COVID-19-associated febrile seizures, approximately 9% of patients required critical care services [22]. The presence of convulsions was recognized as a significant symptom and incorporated into the definition of severe COVID-19 in children elsewhere [13, 14].

The other independent predictors for severity, such as a higher temperature on arrival, vomiting, diarrhea, and rashes, were non-specific when interpreted individually, considering the diversity of presenting symptoms of pediatric COVID-19. Hence, tools that predict the risk of severe COVID-19 in children are required. We constructed a nomogram as a graphical representation of the independent predictive variables. The nomogram portrayed the relative importance of each variable and allowed an individualized risk estimation of severity. Nomograms for predicting the risk of severe COVID-19 from previous studies shared numerous limitations, such as being highly dependent on blood investigations [27, 28], radiological imaging results [29], or designed using a combination of data from children and adults [28, 29], which would greatly hinder their practicality or impact validity attributed to age differences. Conversely, the clinical parameters included in our nomogram were practical and easily obtained, allowing for a quick prediction of moderate/severe disease risk in diverse clinical settings.

The major strength of our study is that it provides statewide data on the epidemiology and clinical manifestations of pediatric COVID-19. This allowed us to document a large spectrum of the presenting features and provided useful information on the disease burden among children in the state. Next, our nomogram demonstrated superior performance characteristics with good discriminatory ability. Our symptom-and-sign-based nomogram provides a practical prediction tool for clinical practice without the need for any laboratory parameters or radiological imaging. Furthermore, our nomogram was based solely on data from children, unlike others that included data from adults and children. Our nomogram would aid in hospitalization decision-making in children, especially at the primary healthcare level or emergency department setting. Children with a predicted probability greater than fifty percent are considered at high risk of moderate/severe disease and should be prioritized for treatment or hospitalization.

This study has several limitations. First, this was a study carried out during a pandemic, and the critical nature of the pandemic only allowed us to obtain baseline symptoms on hospital admission without capturing longitudinal data on the development of new symptoms. The seemingly normal temperature on arrival in both groups of patients could have been explained by antipyretics consumed at home before arriving at the hospital. Second, blood investigations and radiological imaging were only done as clinically indicated to avoid medical resources being overwhelmed by the surge of COVID-19 cases, resulting in incomplete laboratory and radiological data for many patients. Nevertheless, our previous study of pediatric COVID-19 with severe pneumonia found that abnormal biomarkers such as lymphopenia and raised C-reactive protein were typically absent [30]. Third, our nomogram could not separately predict the risk of moderate and severe disease due to the limited number of severe cases. However, our nomogram has clinical utility in predicting the need for hospitalization since all patients with moderate/severe disease require some form of medical intervention during hospitalization. Fourth, the lack of external validation may impact the robustness and performance of the clinical prediction model. Further studies on different populations of children are required to validate our findings. Fifth, genomic sequencing for SARS-CoV-2 variants was not performed in this study. Although the 12-month study duration coincided with the surge of the Delta variant in the country and the rest of the world [9], our findings might not be generalizable to new circulating variants, which might display different clinical outcomes in children.

## Conclusion

Our study provides valuable information on the risk factors for disease severity in pediatric COVID-19. We established a predictive model incorporating parameters that could be easily obtained by clinical assessment. Our nomogram could be a valuable clinical tool for risk stratification to reduce inappropriate hospitalization and identify children at risk of moderate/severe COVID-19 who may benefit from early intervention.

## Supplementary Information

**Additional file 1:** **Supplementary Figure 1. **Model development and internal validation. The data were randomly divided into trainingand validation cohorts. The data from the training cohort were used to train the logistic regression model using the independent factors selected by the multivariate logistic regression analysis. Next, a 5-fold cross-validation was performed on the training set to select optimal model parameters. Finally, the trained model’s performance was evaluated using the validation cohort. The prediction discrimination of the model was calculated as the average values of the area under the curvefor each validation. **Supplementary Figure 2.** Correlation matrix showing correlation coefficients between variables. Values are located between -1 and +1. Values close to -1 are interpreted as negative correlations, and values close to +1 are interpreted as positive correlations. If the coefficient is close to 0, it indicates no correlation between these two variables. Independent variables with a correlation of more than 0·7 were excluded due to the possibility of multicollinearity, but there were no variables that met this criterion. **Supplementary Figure 3.** Trends of hospitalization of pediatric COVID-19 in relation to the % of adults in the state who received two doses of COVID-19 vaccine, January – December 2021. Data for the proportionof the adult population who received COVID-19 vaccination was obtained from the state health department. **Supplementary Figure 4.** The flowchart illustrates the process of selecting variables for inclusion in the final multivariate logistic regression model. Variables that were found to have a *p*-value < 0.05 in the bivariate analysis were included in the multivariate logistic regression model. Finally, variables with a *p*-value greater than 0.05 in the multivariate analysis were excluded from the final model. The variables included in the final model are listed in Table 4.

## Data Availability

All data generated or analysed during this study are included in this published article (and its supplementary information files).

## References

[1] Gotzinger F, Santiago-Garcia B, Noguera-Julian A, Lanaspa M, Lancella L, Calo Carducci FI (2020). COVID-19 in children and adolescents in Europe: a multinational, multicentre cohort study. Lancet Child Adolesc Health.

[2] Tagarro A, Cobos-Carrascosa E, Villaverde S, Sanz-Santaeufemia FJ, Grasa C, Soriano-Arandes A (2022). Clinical spectrum of COVID-19 and risk factors associated with severity in Spanish children. Eur J Pediatr.

[3] Antoon JW, Grijalva CG, Thurm C, Richardson T, Spaulding AB, Teufel RJ (2021). Factors Associated With COVID-19 Disease Severity in US Children and Adolescents. J Hosp Med.

[4] Woodruff RC, Campbell AP, Taylor CA, Chai SJ, Kawasaki B, Meek J (2021). Risk Factors for Severe COVID-19 in Children. Pediatrics.

[5] Bellino S, Punzo O, Rota MC, Del Manso M, Urdiales AM, Andrianou X (2020). COVID-19 Disease Severity Risk Factors for Pediatric Patients in Italy. Pediatrics.

[6] Malaysia MOH. Management of Suspected, Probable and Confirmed COVID-19 Case in Malaysia. 2022. Available at: https://covid-19.moh.gov.my/garis-panduan/garis-panduan-kkm/ANNEX-2-Management-of-Suspected-Probable-and-Confirmed-COVID19-05042022.pdf. Accessed 18 Sept 2022.

[7] Centers for Disease Control and Prevention. Defining Child BMI Categories. Available at: https://www.cdc.gov/obesity/childhood/defining.html. Accessed 18 Sept 2022.

[8] Wg WHO. A minimal common outcome measure set for COVID-19 clinical research. Lancet Infect Dis. 2020;20(8):e192–7. Available at: 10.1016/S1473-3099(20)30483-7. Accessed 18 Sept 2022. 10.1016/S1473-3099(20)30483-7 PMC7292605 32539990

[9] Hodcroft E. CoVariants : Overview of Variants in Countries. 2022. https://covariants.org/per-country.

[10] Ng DC, Tan KK, Chin L, Cheng XL, Vijayakulasingam T, Liew DWX (2022). Risk factors associated with household transmission of SARS-CoV-2 in Negeri Sembilan. Malaysia J Paediatr Child Health.

[11] Dong Y, Mo X, Hu Y, Qi X, Jiang F, Jiang Z (2020). Epidemiology of COVID-19 Among Children in China. Pediatrics.

[12] Parri N, Magista AM, Marchetti F, Cantoni B, Arrighini A, Romanengo M (2020). Characteristic of COVID-19 infection in pediatric patients: early findings from two Italian Pediatric Research Networks. Eur J Pediatr.

[13] Wu Q, Xing Y, Shi L, Li W, Gao Y, Pan S (2020). Coinfection and Other Clinical Characteristics of COVID-19 in Children. Pediatrics.

[14] Qiu H, Wu J, Hong L, Luo Y, Song Q, Chen D (2020). Clinical and epidemiological features of 36 children with coronavirus disease 2019 (COVID-19) in Zhejiang, China: an observational cohort study. Lancet Infect Dis.

[15] Preston LE, Chevinsky JR, Kompaniyets L, Lavery AM, Kimball A, Boehmer TK (2021). Characteristics and Disease Severity of US Children and Adolescents Diagnosed With COVID-19. JAMA Netw Open.

[16] Swann OV, Holden KA, Turtle L, Pollock L, Fairfield CJ, Drake TM (2020). Clinical characteristics of children and young people admitted to hospital with covid-19 in United Kingdom: prospective multicentre observational cohort study. BMJ.

[17] Chao JY, Derespina KR, Herold BC, Goldman DL, Aldrich M, Weingarten J (2020). Clinical Characteristics and Outcomes of Hospitalized and Critically Ill Children and Adolescents with Coronavirus Disease 2019 at a Tertiary Care Medical Center in New York City. J Pediatr.

[18] Bhopal SS, Bagaria J, Olabi B, Bhopal R (2021). Children and young people remain at low risk of COVID-19 mortality. Lancet Child Adolesc Health.

[19] Wong JJM, Abbas Q, Chuah SL, Malisie RF, Pon KM, Katsuta T (2021). Comparative Analysis of Pediatric COVID-19 Infection in Southeast Asia, South Asia, Japan, and China. Am J Trop Med Hyg.

[20] Tan L, Ganapathy SS, Chan YM, Alias N, Nasaruddin NH, Khaw WF (2022). Estimating the COVID-19 mortality burden over two full years of the pandemic in Malaysia. Lancet Reg Health West Pac.

[21] Sayed IA, Bhalala U, Strom L, Tripathi S, Kim JS, Michaud K (2022). Gastrointestinal Manifestations in Hospitalized Children With Acute SARS-CoV-2 Infection and Multisystem Inflammatory Condition: An Analysis of the VIRUS COVID-19 Registry. Pediatr Infect Dis J.

[22] Cadet K, Boegner J, Ceneviva GD, Thomas NJ, Krawiec C (2022). Evaluation of Febrile Seizure Diagnoses Associated With COVID-19. J Child Neurol.

[23] Ng DC, Tan KK, Chin L, Ali MM, Lee ML, Mahmood FM (2021). Clinical and epidemiological characteristics of children with COVID-19 in Negeri Sembilan. Malaysia Int J Infect Dis.

[24] Brewster RC, Parsons C, Laird-Gion J, Hilker S, Irwin M, Sommerschield A (2022). COVID-19-Associated Croup in Children. Pediatrics.

[25] Graff K, Smith C, Silveira L, Jung S, Curran-Hays S, Jarjour J (2021). Risk Factors for Severe COVID-19 in Children. Pediatr Infect Dis J.

[26] Oliveira EA, Colosimo EA, Simoes ESAC, Mak RH, Martelli DB, Silva LR (2021). Clinical characteristics and risk factors for death among hospitalised children and adolescents with COVID-19 in Brazil: an analysis of a nationwide database. Lancet Child Adolesc Health.

[27] Zhou B, Yuan Y, Wang S, Zhang Z, Yang M, Deng X (2021). Risk profiles of severe illness in children with COVID-19: a meta-analysis of individual patients. Pediatr Res.

[28] Oh B, Hwangbo S, Jung T, Min K, Lee C, Apio C (2021). Prediction Models for the Clinical Severity of Patients With COVID-19 in Korea: Retrospective Multicenter Cohort Study. J Med Internet Res.

[29] Yu Y, Wang X, Li M, Gu L, Xie Z, Gu W (2020). Nomogram to identify severe coronavirus disease 2019 (COVID-19) based on initial clinical and CT characteristics: a multi-center study. BMC Med Imaging.

[30] Ng DC, Tan KK, Ting GSS, Ling C, Fadzilah NFB, Tan SF (2022). Comparison of Severe Viral Pneumonia Caused by SARS-CoV-2 and Other Respiratory Viruses Among Malaysian Children During the COVID-19 Pandemic. Front Pediatr.

