# Supplementary material for: Risk factors for disease severity among children with Covid-19: a clinical prediction model
Source: BMC Infect Dis. 2023 Jun 12;23:398. doi: 10.1186/s12879-023-08357-y (PMC10259359; doi:10.1186/s12879-023-08357-y)
Supplement: Supplementary file 1 — Additional file 1: Supplementary Figure 1. Model development and internal validation. The data were randomly divided into trainingand validation cohorts. The data from the training cohort were used to train the logistic regression model using the independent factors selected by the multivariate logistic regression analysis. Next, a 5-fold cross-validation was performed on the training set to select optimal model parameters. Finally, the trained model’s performance was evaluated using the validation cohort. The prediction discrimination of the model was calculated as the average values of the area under the curvefor each validation. Supplementary Figure 2. Correlation matrix showing correlation coefficients between variables. Values are located between -1 and +1. Values close to -1 are interpreted as negative correlations, and values close to +1 are interpreted as positive correlations. If the coefficient is close to 0, it indicates no correlation between these two variables. Independent variables with a correlation of more than 0·7 were excluded due to the possibility of multicollinearity, but there were no variables that met this criterion. Supplementary Figure 3. Trends of hospitalization of pediatric COVID-19 in relation to the % of adults in the state who received two doses of COVID-19 vaccine, January – December 2021. Data for the proportionof the adult population who received COVID-19 vaccination was obtained from the state health department. Supplementary Figure 4. The flowchart illustrates the process of selecting variables for inclusion in the final multivariate logistic regression model. Variables that were found to have a p-value < 0.05 in the bivariate analysis were included in the multivariate logistic regression model. Finally, variables with a p-value greater than 0.05 in the multivariate analysis were excluded from the final model. The variables included in the final model are listed in Table 4. [file 12879_2023_8357_MOESM1_ESM.docx]

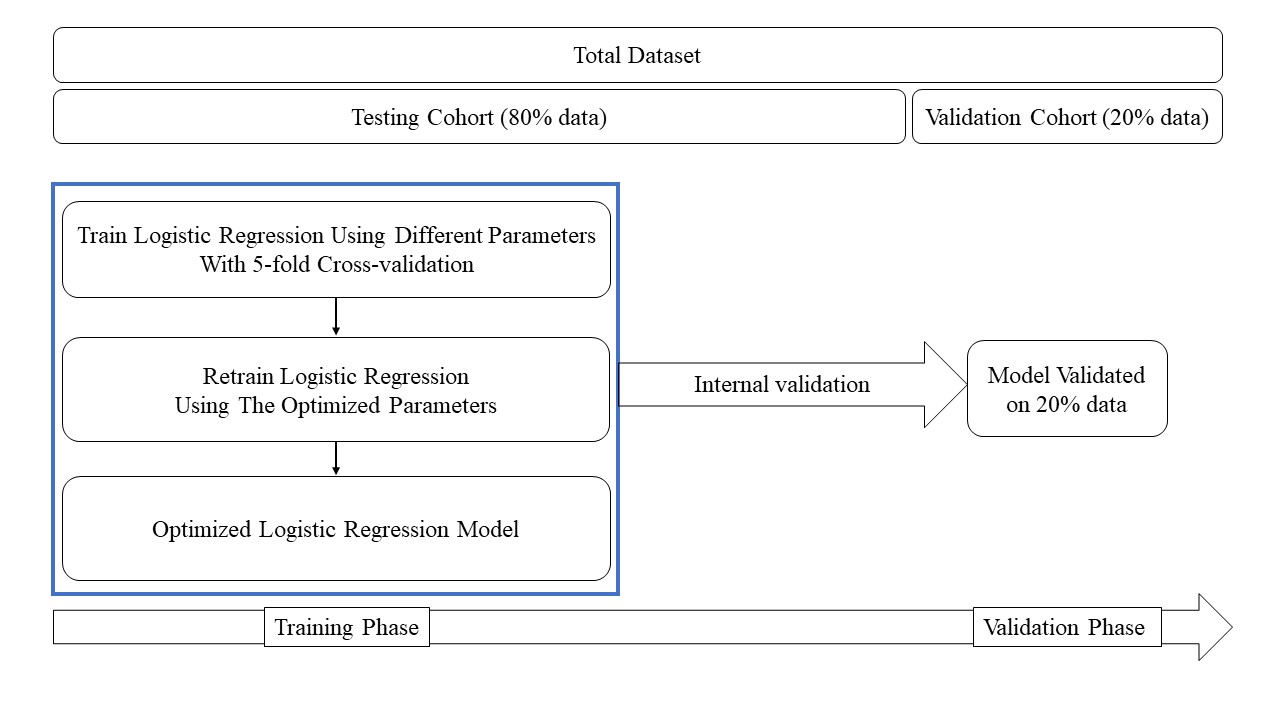
 **Supplementary Figure 1:** Model development and internal validation. The data were randomly divided into training (80%) and validation cohorts (20%). The data from the training cohort were used to train ­­the logistic regression model using the independent factors selected by the multivariate logistic regression analysis. Next, a 5-fold cross-validation was performed on the training set to select optimal model parameters. Finally, the trained model’s performance was evaluated using the validation cohort. The prediction discrimination of the model was calculated as the average values of the area under the curve (AUC) for each validation.

**Supplementary Figure 2:** Correlation matrix showing correlation coefficients between variables. Values are located between -1 and +1. Values close to -1 are interpreted as negative correlations, and values close to +1 are interpreted as positive correlations. If the coefficient is close to 0, it indicates no correlation between these two variables. Independent variables with a correlation of more than 0·7 were excluded due to the possibility of multicollinearity, but there were no variables that met this criterion.


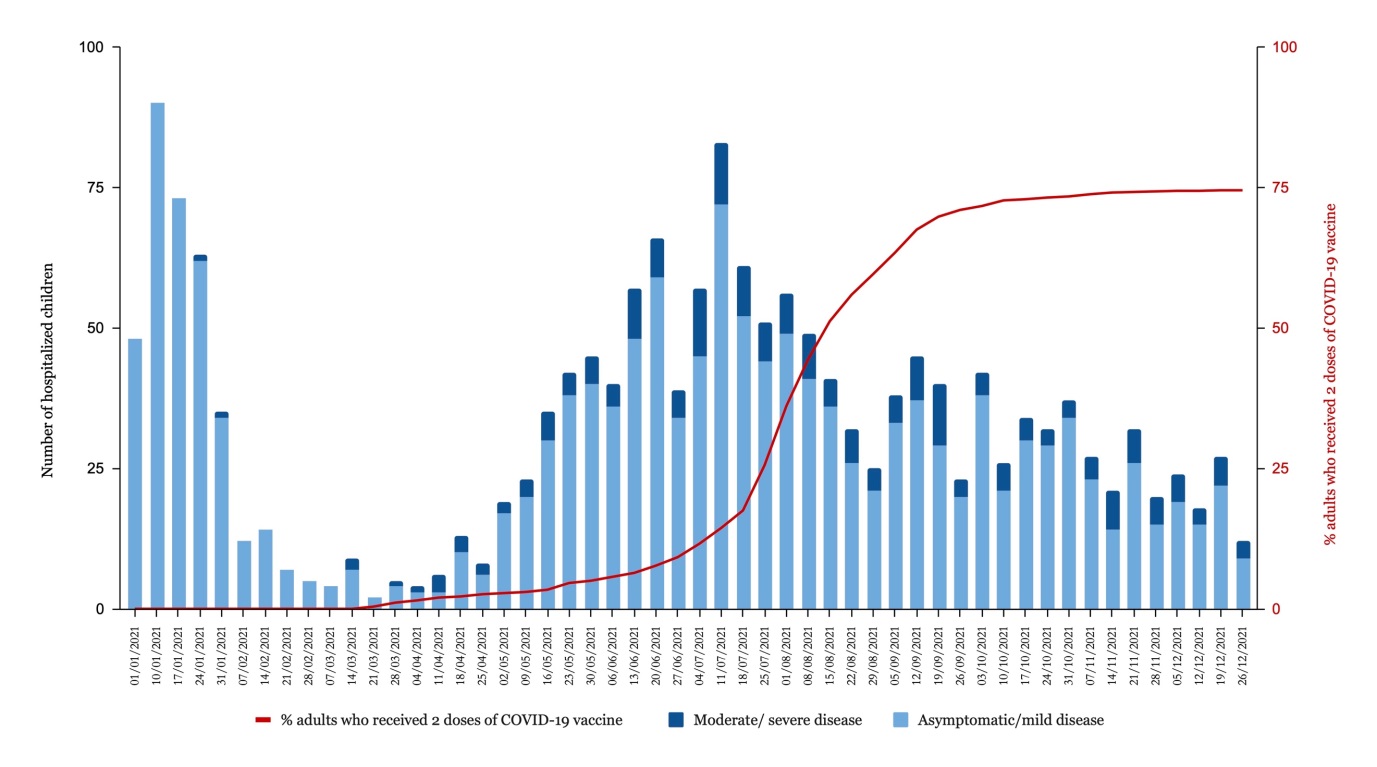


**Supplementary Figure 3:** Trends of hospitalization of pediatric COVID-19 in relation to the % of adults in the state who received two doses of COVID-19 vaccine, January – December 2021. Data for the proportion (%) of the adult population who received COVID-19 vaccination was obtained from the state health department.


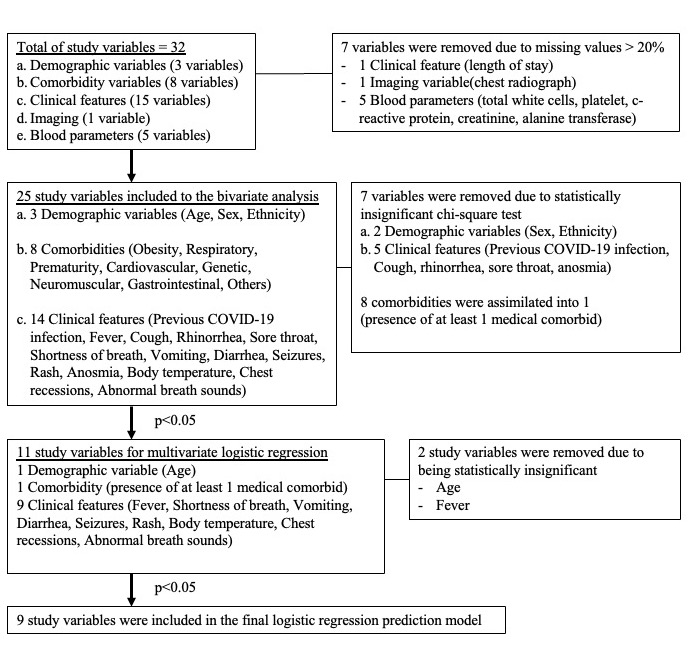


**Supplementary Figure 4:** The flowchart illustrates the process of selecting variables for inclusion in the final multivariate logistic regression model. Variables that were found to have a p-value < 0.05 in the bivariate analysis were included in the multivariate logistic regression model. Finally, variables with a p-value greater than 0.05 in the multivariate analysis were excluded from the final model. The variables included in the final model are listed in Table 4.
